# Prediction of MoRFs in Protein Sequences with MLPs Based on Sequence Properties and Evolution Information

**DOI:** 10.3390/e21070635

**Published:** 2019-06-27

**Authors:** Hao He, Jiaxiang Zhao, Guiling Sun

**Affiliations:** College of Electronic Information and Optical Engineering, Nankai University, Tianjin 300350, China

**Keywords:** molecular recognition features, intrinsically disordered proteins, multi-layer perceptron

## Abstract

Molecular recognition features (MoRFs) are one important type of intrinsically disordered proteins functional regions that can undergo a disorder-to-order transition through binding to their interaction partners. Prediction of MoRFs is crucial, as the functions of MoRFs are associated with many diseases and can therefore become the potential drug targets. In this paper, a method of predicting MoRFs is developed based on the sequence properties and evolutionary information. To this end, we design two distinct multi-layer perceptron (MLP) neural networks and present a procedure to train them. We develop a preprocessing process which exploits different sizes of sliding windows to capture various properties related to MoRFs. We then use the Bayes rule together with the outputs of two trained MLP neural networks to predict MoRFs. In comparison to several state-of-the-art methods, the simulation results show that our method is competitive.

## 1. Introduction

Intrinsically disordered proteins (IDPs) possess flexible and instable structures which make them play a crucial role in a variety of important biological functions [1]. Being an important type of functional region in IDPs, molecular recognition features (MoRFs), generally consisting of 10–70 consecutive residues and are located in the long disordered regions, can undergo a disorder-to-order transition through binding to their interaction partners [2,3]. There are four types of MoRFs, i.e., *α*-MoRFs, *β*-MoRFs, *γ*-MoRFs and complex-MoRFs, which correspond to *α*-helix, *β*-strand, coil secondary structures and multiple secondary structures [4]. Many MoRFs, acting as molecular switches in molecular-interaction networks, play a role in the signaling and alternative splicing of cells [2]. It is observable that MoRFs are abundant in proteins with recognition functions [5]. Prediction of MoRFs is crucial, as the functions of MoRFs are associated with many diseases and may therefore be potential drug targets [6].

In recent years, many computational schemes for predicting MoRFs have been reported, including α-MoRF-Pred I [5], α-MoRF-Pred II [7], ANCHOR [8], MoRFpred [9], MSPSSMpred [10], DISOPRED3 [11] and MoRF_CHiBi_ [12]. Of these reported methods, α-MoRF-Pred I and α-MoRF-Pred II are only capable of predicting α-MoRFs. ANCHOR, using estimated energy calculations [13] to capture the biophysical properties of MoRFs, cannot yield enough prediction accuracy in many cases. MoRFpred is a comprehensive method relying on a lot of features, such as evolutionary information [14] and physiochemical properties [15], solvent accessibility [16], and B-factors [17], as well as predicted disorder probabilities from several other predictors [18,19,20,21]. MoRF_CHiBi_ predicts MoRFs by extracting features from physicochemical properties [15] and utilizing two support vector machine (SVM) models [12]. MoRF_CHiBi_ does not depend on the results of other predictors, and obtains better prediction performance than MoRFpred. However, the prediction accuracy of MoRF_CHiBi_ is still expected to improve. Besides, MoRF_CHiBi_Web_ [22] and MoRF_CHiBi_Light_ [23] are two methods based on combining MoRF_CHiBi_ with other predictors, and obtain better performance than MoRF_CHiBi_. OPAL [24] is also a combined predictor, which utilizes the results of two independent predictors. The first one is MoRF_CHiBi_, and the second one is PROMIS [24], obtained by half-sphere exposure, solvent accessible surface area and backbone angle information. These combined predictors usually perform better than individual predictors. However, they all need to combine MoRF_CHiBi_ with other predictors. 

In this paper, we design an individual prediction method for MoRFs based on two distinct multi-layer perceptron (MLP) neural networks where one of them is MLP1 trained by the 16 sequence properties and the other is MLP2 with the evolutionary information. We present a procedure to train these two MLP neural networks. The training procedure utilizes the data from the preprocessing process developed by us, which involves different sizes of the sliding windows to capture various properties related to MoRFs. The outputs of MLP1 and MLP2 are then used to predict MoRFs based on the Bayes rule. Finally, the TEST464 and TEST_EXP53 sets are used to compare the performances of our method with ANCHOR, MoRFpred, MoRF_CHiBi_, MoRF_CHiBi_Web_, MoRF_CHiBi_Light_, PROMIS and OPAL. The simulation results show that the AUCs generated from our method are equal to 0.806 and 0.792 on the TEST464 and TEST_EXP53 sets, respectively.

## 2. Materials and Methods

In this section, we employ the sequence properties as well as the evolutionary information of the protein sequence to train two distinct MLP neural networks respectively. Utilizing the probability distributions yielded from these two distinct MLP neural networks, we then follow the Bayes rule to predict MoRFs.

### 2.1. Datasets

For comparison with other methods, we use the same datasets created by Disfani et al. [9], which is from Protein Data Bank (PDB) [25]. The datasets include 840 MoRFs, which contain 181 helical, 34 strand, 595 coil and 30 complex MoRF regions. In [9], the 840 MoRFs dataset are divided into the TRAINING and TEST set with which there are 421 and 419 protein sequences respectively. Thus, the TRAINING set contains 245,984 residues including 5396 MoRF residues, while the TEST set has 258,829 residues with 5153 MoRF residues. After that, Disfani et al. also used another test set, TESTNEW. TESTNEW has 45 sequences consisting of 37,533 residues with 626 MoRF residues. We combine the TEST and TESTNEW sets into single set TEST464. In addition, we use the TEST_EXP53 assembled by Malhis et al. [22] as the third test set. TEST_EXP53 has 53 sequences, including 2432 MoRF residues, which consist of 729 short MoRF residues (up to 30 residues) and 1703 long MoRF residues (more than 30 residues). We use the same TRAINING set to train our two distinct MLP neural networks and TEST set to evaluate it. The TEST464 and TEST_EXP53 sets are utilized to compare our method with other methods.

### 2.2. Feature Selection

We rely on the sequence properties and evolutionary information of the protein sequences to predict MoRFs. Protein sequences with MoRFs generally comprise of MoRFs, MoRFs’ flanking (Flanks) and other non-MoRF residues. We define the Flanks as other residues in the disordered regions where MoRFs are located. Our computation of the sequence properties and evolutionary information of the protein sequences does not require to do any special treatment on Flanks relies only on the protein sequences.

The sequence properties we use include 13 physicochemical properties from the Amino Acid Index [15] listed in Table A1 of Appendix A and 3 structural properties, which include the topological entropy [26], and the Remark 465 and Deleage/Roux propensities, both from the GlobPlot NAR paper [27]. Evolutionary information in this paper is obtained by the Position Specific Scoring Matrix (PSSM) through executing three iterations of PSI-BLAST against NCBI [14] non-redundant database with default parameters.

The 16 sequence properties we select are preprocessed as follows: Given a protein sequence of length L, we select a sliding window of size N(N<L) and append ⎣N/2⎦ zeros to both ends of the sequence. In each region determined by the window, we compute the topological entropy through Equation 14 of [26]. Each of the rest 15 sequence properties is assigned to the average value of the amino acid scales of the residues in this region. Thus, each window can obtain a 16-dimensional vector $v_{i}\left(1\leq i\leq L\right)$. Then, we associate it with every residue in the window. Finally, for each residue, we assign the average value of all the $v_{i}$ associated with it as the feature vector of this specific residue. The feature vector $x_{j}\left(1\leq j\leq L\right)$ can be computed as

(1) $$x_{j}=\{\begin{array}{c} \frac{1}{j+N_{0}}\overset{j+N_{0}}{\underset{i=1}{\sum }}v_{i},1\leq j\leq N_{0}\\ \frac{1}{N}\overset{j+N_{0}}{\underset{i=j+N_{0}-N+1}{\sum }}v_{i},N_{0}<j\leq L-N_{0}\\ \frac{1}{L_{0}-j-N_{0}+1}\overset{L_{0}-N+1}{\underset{i=j+N_{0}-N+1}{\sum }}v_{i},L-N_{0}<j\leq L \end{array},$$

where $N_{0}=⎣N/2⎦$ and $L_{0}=L+2N_{0}$. In this paper, we use three sizes of the sliding windows, i.e., *N* = 10, 45 and 90, to compute 16 sequence properties. The smaller size of the sliding window is used to capture properties especially related to MoRFs, as they are usually much shorter than the surrounding disordered regions. The longer sliding windows are used to extract information from the surrounding regions of MoRFs. Thus, using these three sizes of sliding windows, we can compute a 48-dimensional feature vector associated with each residue of the protein sequence. 

For this given protein sequence of length L, the evolutionary information is computed through the PSSM which yields a 20×L matrix [28]. We first transform this 20×L matrix into a $20\times L_{0}$ matrix by appending 20×⎣N/2⎦ zeros at the beginning and end of this 20×L matrix, respectively. Then we again choose three sizes of the sliding windows, i.e., *N* = 10, 45 and 90, to slice a 20×N matrix from the $20\times L_{0}$ transformed matrix. We can compute an average value for each row for this 20×N matrix, and then use Equation (1) to yield a 20×1 vector for each residue. Since three sizes of the sliding windows are employed, a 60-dimensional feature vector containing the evolutionary information is obtained for each residue.

### 2.3. MLP Prediction Models

We train two distinct multi-layer perceptron (MLP) neural networks, where one of them is trained using the sequence properties and the other is trained through the evolutionary information obtained from the above section. Both these models contain two hidden layers, with each hidden layer having 12 perceptrons and one bias. We use the ReLU functions as the activation functions in each hidden layer and the sigmoid functions in the output layers. During the training process, a dropout algorithm [29] is utilized to avoid overfitting. The forward propagation with dropout algorithm proceeds as follows:

(2) $$Z^{\left[l\right]}=W^{\left[l\right]}\cdot A^{\left[l-1\right]}+b^{\left[l\right]},$$

(3) $$A^{\left[l\right]}=g^{\left[l\right]}\left(Z^{\left[l\right]}\right)*R\left(p_{d}\right)\;,$$

where $g^{\left[l\right]}$ denotes the vector activation function of the l-th layer, and l=1,2,3 in our model. $R\left(p_{d}\right)$ is a vector obeying the Bernoulli distribution with $p_{d}$ being the dropout parameter which represents the remaining probability of each perceptron in the hidden layers. Furthermore, $A^{\left[0\right]}$ is the input feature matrix and $A^{\left[3\right]}$ is the prediction result. Then, we employ the Adam algorithm [30] to optimize $W^{\left[l\right]}$ and $b^{\left[l\right]}$ in the back propagation.

**Figure 1:**
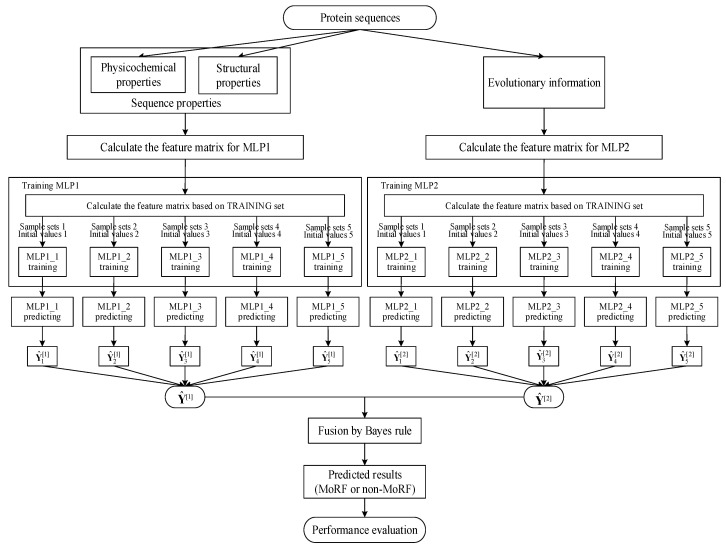
Detailed paradigm of the proposed method.

From Section 2.1, the TRAINING set contains 245,984 residues, among which there are 5396 MoRF residues. We only randomly select 5396 non-MoRF residues from the TRAINING set to train our two MLP neural networks, which ensures our trained MLP neural networks to being capable of more effectively identifying both MoRF and non-MoRF residues. Finally, in order to increase the robust of our MLP neural networks and reduce the influence of the initial weights, we train the two MLP neural networks five times and use the average values of them as the final outputs of our MLP neural networks. Finally, we use the Bayes rule together with the outputs of our two MLP neural networks to compute the prediction of MoRFs. The detailed paradigm of our method is shown in Figure 1.

### 2.4. Performance Evaluation

We use the ROC curve and three evaluation metrics to evaluate performance in this paper. These are the AUC (the area under the ROC curve), TPR (the true positive rate) and FPR (the false positive rate). The computation equations of TPR and FPR are $\mathrm{TPR}=TP/N_{\mathrm{MoRF}}\;,\;\mathrm{FPR}=TN/N_{\mathrm{non}}$, where TP and TN respectively represent the numbers of accurately predicted MoRFs and non-MoRFs residues. In addition, we denote the total number of MoRFs and non-MoRFs residues as *N*_MoRF_ and *N*_non_, respectively. 

## 3. Results and Discussion

Using the TEST set defined in Section 2.1, we run our trained multi-layer perceptron (MLP) neural networks. The outputs of two trained MLP neural networks MLP1 and MLP2 are then utilized to predict MoRFs based on the Bayes rule. Finally, the TEST464 and TEST_EXP53 sets are used to compare the performances of our method with ANCHOR, MoRFpred, MoRF_CHiBi_, MoRF_CHiBi_Web_, MoRF_CHiBi_Light_, PROMIS and OPAL. The simulation results show that the AUC values generated from our method are equal to 0.806 and 0.792 on the TEST464 and TEST_EXP53 sets, respectively.

### 3.1. Prediction Performance of Sequence Properties

**Figure 2:**
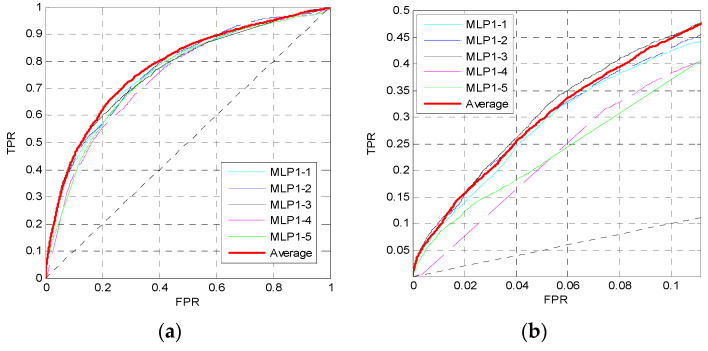
ROC curves of MLP1 on the TEST set. (**a**) The overall ROC curves. (**b**) The ROC curves at low FPR region.

For the 16 sequence properties, three windows with lengths of 10, 45 and 90 are used to perform preprocessing and calculate the feature matrix. Then we train the first MLP neural network MLP1 as shown in Figure 1. In MLP1, the perceptron number of two hidden layers is set to *N*_neur_ = [12,12], where the two numbers correspond to the perceptron numbers of the first hidden layer and the second hidden layer. The dropout parameter is *p_d_* = 0.5, and the learning rate is 0.001. Figure 2 shows the ROC curves of five independent MLPs of MLP1 on the TEST set, and the ROC curves of the average values which are described by the red curves. Figure 2a shows the overall ROC curves, and Figure 2b shows the ROC curves in the low FPR region.

From Figure 2a, the red curve is higher than the other curves. Furthermore, in Figure 2b, although the pink curve is the highest, the red one is very close to it, and the pink curve is obviously lower than the red one when FPR>0.2. Similar to the pink curve, other curves may be slightly higher than the red curve in some small regions, but may be lower in other regions. Therefore, the prediction performance can be improved by training five independent networks and taking their average values of outputs as the final outputs of the MLP1. 

### 3.2. Impact of Different MLP1 Parameters

**Figure 3:**
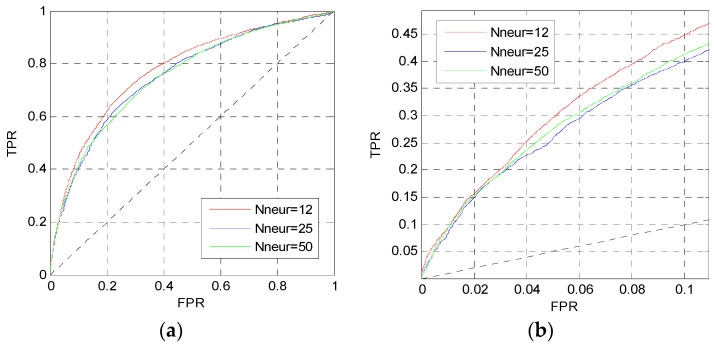
ROC curves of MLP1 with different *N*_neur_. (**a**) The overall ROC curves; (**b**) the ROC curves in the low FPR region.

In this section, we change the perceptron number *N*_neur_ and the dropout parameter *p_d_* in MLP1 to analysis their influence. Firstly, we change the perceptron number, and set the dropout parameter and the learning rate to 0.5 and 0.001, respectively. Figure 3 shows the ROC curves of MLP1 on the TEST set when *N*_neur_ = [12,12], [25,25], [50,50]. 

The curves in Figure 3 are the prediction results of MLP1 calculated by the average values of five independent networks. The red curves are higher than other curves in Figure 3a,b. Thus, the perceptron number of MLP1 is set to *N*_neur_ = [12,12].

**Figure 4:**
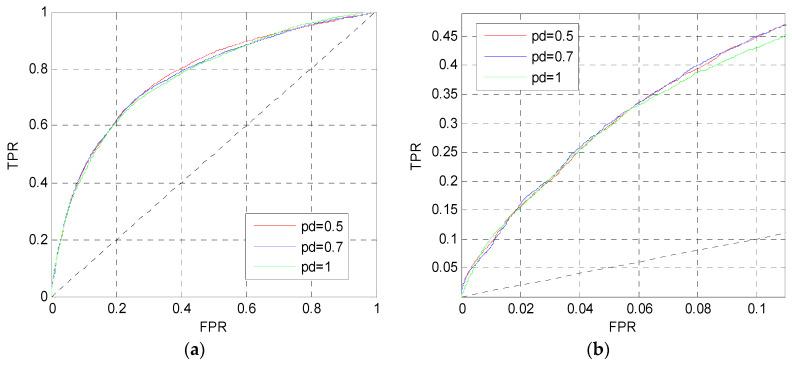
ROC curves of MLP1 with different *p_d_*. (**a**) The overall ROC curves. (**b**) The ROC curves in the low FPR region.

After determining the perceptron number, we change the dropout parameter *p_d_* in MLP1. Figure 4 shows the ROC curves on the TEST set when *p_d_* = 0.5, 0.7, 1. Although these three curves are approximate to each other in Figure 4a,b, the red curve in Figure 4a is slightly higher than the others, and the red and blue curves in Figure 4b are slightly higher than the pink curve. Finally, the dropout parameter of MLP1 is set to *p_d_* = 0.5.

### 3.3. Prediction Performance of Evolutionary Information

**Figure 5:**
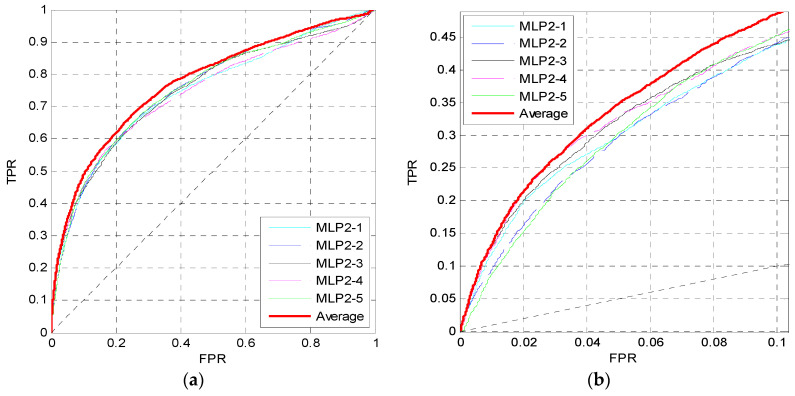
ROC curves of MLP2 on the TEST set. (**a**) The overall ROC curves; (**b**) the ROC curves at low FPR region.

For the evolutionary information, after calculating the PSSM for each protein sequence, three windows of 10, 45 and 90 are also used to perform preprocessing and calculate the feature matrix. Then we train the second MLP neural network MLP2 as shown in Figure 1. We first set the perceptron number to *N*_neur_ = [25,25] in MLP2. The dropout parameter is *p_d_* = 0.7, and the learning rate is 0.0001. Similar to Figure 2, Figure 5 shows the ROC curves of five independent MLPs of MLP2 on the TEST set, and the ROC curves of the average values, which are also the red curves. 

From Figure 5, the red curves are higher than other curves both in (a) and (b). Thus, for the evolutionary information, the prediction performance is also improved by training five independent networks and taking their average values of outputs as the final outputs of MLP2.

### 3.4. Impact of Different MLP2 Parameters

**Figure 6:**
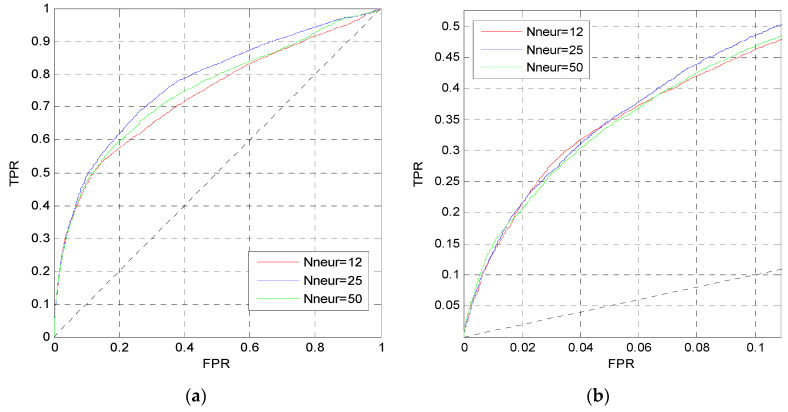
ROC curves of MLP2 with different *N*_neur_. (**a**) The overall ROC curves. (**b**) The ROC curves in the low FPR region.

We change the perceptron number *N*_neur_ and the dropout parameter *p_d_* in MLP2 to analyze their influence. Firstly, we only change *N*_neur_; simultaneously, the dropout parameter is set to *p_d_* = 0.7 and the learning rate is also 0.0001. Figure 6 shows the ROC curves of MLP2 on the TEST set when *N*_neur_ = [12,12], [25,25], [50,50]. In Figure 6a, the blue curve is higher than the other curves, and it is also higher when FPR > 0.06 in Figure 6b. Finally, we set *N*_neur_ = [25,25] in MLP2.

**Figure 7:**
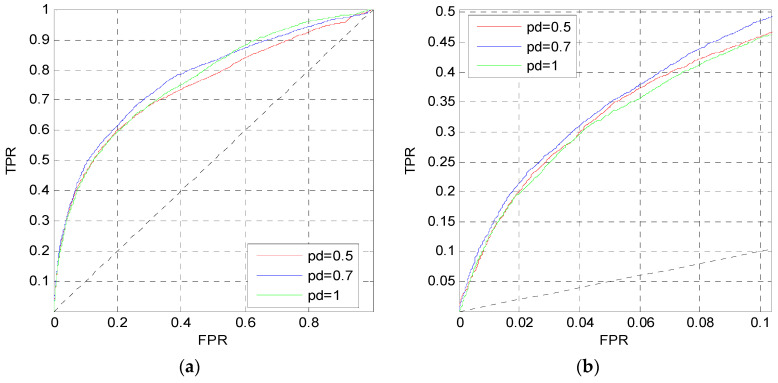
ROC curves of MLP2 with different *p_d_*. (**a**) The overall ROC curves. (**b**) The ROC curves in the low FPR region.

After determining the perceptron number in MLP2, we change the dropout parameter *p_d_*. Figure 7 shows the ROC curves on the TEST set when *p_d_* = 0.5, 0.7, 1. In Figure 7a, the blue and pink curves are better than the red curve. However, the blue curve is better than the other curves in Figure 7b. Therefore, the dropout parameter of MLP2 is set to *p_d_* = 0.7.

### 3.5. Prediction Performance of the Fusion Results

**Figure 8:**
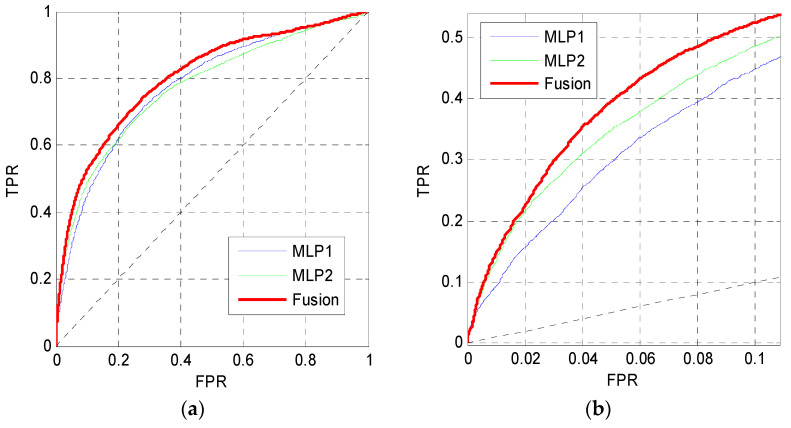
ROC curves of MLP1, MLP2 and the fusion results. (**a**) The overall ROC curves. (**b**) The ROC curves at low FPR region.

In this section, the outputs of MLP1 and MLP2 are fused using the Bayes rule to predict MoRFs. Figure 8 shows the ROC curves of MLP1, MLP2 and the fusion results on the TEST set. The ROC curve of MLP2 gets a better performance in the low FPR region, while the ROC curve of MLP1 performs better when the FPR is higher than 0.2. Thus, the ROC curves of MLP1 and MLP2 cross each other in Figure 8a. However, the curve of the fusion results is higher than the other curves both in low FPR and high FPR regions.

### 3.6. Comparison with other Methods

In this section, using the TEST464 and TEST_EXP53 sets, we compare our method, named MoRF_MLP_, with ANCHOR, MoRFpred, MoRF_CHiBi_, MoRF_CHiBi_Web_, MoRF_CHiBi_Light_, PROMIS and OPAL. The results of other methods are from [24] and the online predictor of MoRF_CHiBi_ system. Table 1 shows the AUC values of these methods that run on these two test sets. Since TEST_EXP53 contains long MoRF regions, we not only compare the AUC values on the overall dataset (EXP53_all), but also compare the AUC values on the datasets that only contain long MoRF regions (EXP53_long) and short MoRF regions (EXP53_short), respectively. In these methods, MoRF_CHiBi_Web_, MoRF_CHiBi_Light_ and OPAL are combined component predictors. They usually perform better than individual predictors. The bold data in Table 1 indicate the best values in individual and combined component predictors, respectively. Our method is an individual predictor, so we mainly compare with ANCHOR, MoRFpred, MoRF_CHiBi_ and PROMIS, which are also individual predictors. From Table 1, MoRF_MLP_ obtains a higher AUC on the TEST464 set, and PROMIS gets a higher AUC on the TEST_EXP53 set. 

In addition, to further analyze the prediction performance of these methods, we also calculate the FPR values at different TPR on TEST464 and EXP53_all sets, as shown in Table 2. From Table 2, MoRF_MLP_ gets the lowest FPR when TPR is set to 0.2, 0.3 and 0.4 in five individual predictors, which indicates that MoRF_MLP_ can obtain higher TPR at low FPR. Therefore, as an individual predictor, MoRF_MLP_ is competitive.

## 4. Conclusions

In this paper, we propose a new method, MoRF_MLP_, to predict MoRFs. We employ the sequence properties as well as the evolutionary information to train two distinct MLP neural networks. The sequence properties contain 13 physicochemical properties and 3 structural properties, and are extracted by preprocessing using 3 different windows. The evolutionary information is extracted from PSSM and preprocessed by the same windows as sequence properties. Then, the outputs of the two MLP neural networks are utilized to predict MoRFs based on Bayes rule. Finally, we test MoRF_MLP_ using TEST464 and TEST_EXP53 sets. Compared to other individual predictors, the simulation results show that MoRF_MLP_ achieves higher AUC on TEST464 set, and gets higher TPR at low FPR on TEST464 and EXP53_all sets.

## Figures and Tables

**Table 1:** AUC on TEST464 and TEST_EXP53.

	TEST464	EXP53_All	EXP53_Short	EXP53_Long
MoRF_MLP_	**0.806**	0.792	0.818	0.781
ANCHOR	0.605	0.615	0.683	0.586
MoRFpred	0.675	0.620	0.673	0.598
PROMIS	0.790	**0.818**	**0.823**	**0.815**
MoRF_CHiBi_	0.743	0.712	0.790	0.679
MoRF_CHiBi_Light_,	0.777	0.799	0.869	0.770
MoRF_CHiBi_Web_	0.805	0.797	**0.886**	0.758
OPAL	**0.816**	**0.836**	0.870	**0.823**

**Table 2:** FPR at different TPR on TEST464 and EXP53_all.

	TPR = 0.2		TPR = 0.3		TPR = 0.4	
	TEST464	EXP53_All	TEST464	EXP53_All	TEST464	EXP53_All
MoRF_MLP_	**0.015**	**0.030**	**0.029**	**0.051**	**0.051**	**0.079**
ANCHOR	0.079	0.104	0.163	0.173	0.246	0.263
MoRFpred	0.033	0.083	0.071	0.146	0.143	0.221
PROMIS	0.031	0.032	0.069	0.056	0.103	0.081
MoRF_CHiBi_	0.031	0.031	0.063	0.064	0.104	0.125
MoRF_CHiBi_Light_,	0.020	0.016	0.040	0.043	0.073	0.068
MoRF_CHiBi_Web_	**0.016**	0.016	**0.033**	0.033	**0.057**	0.061
OPAL	0.025	**0.015**	0.052	**0.029**	0.074	**0.056**

## Appendix A

The 13 physicochemical properties selected from the Amino Acid Index are given in the following list.

**Table A1:** The properties selected from the Amino Acid Index.

Index:	Definition:
CIDH920101	Normalized hydrophobicity scales for alpha-proteins
EISD860103	Direction of hydrophobic moment
NISK860101	14 A contact number
QIAN880105	Weights for alpha-helix at the window position of -2
ROBB760101	Information measure for alpha-helix
ROBB760108	Information measure for turn
ROBB760112	Information measure for coil
ROBB760113	Information measure for loop
CORJ870103	PRIFT index
CORJ870106	ALTLS index
CORJ870107	TOTFT index
CORJ870108	TOTLS index
MIYS990104	Optimized relative partition energies—method C
